# TAM family receptors in conjunction with MAPK signalling are involved in acquired resistance to PI3Kα inhibition in head and neck squamous cell carcinoma

**DOI:** 10.1186/s13046-020-01713-9

**Published:** 2020-10-15

**Authors:** Kara M. Ruicci, Jalna Meens, Paul Plantinga, William Stecho, Nicole Pinto, John Yoo, Kevin Fung, Danielle MacNeil, Joe S. Mymryk, John W. Barrett, Christopher J. Howlett, Paul C. Boutros, Laurie Ailles, Anthony C. Nichols

**Affiliations:** 1 Department of Otolaryngology - Head and Neck Surgery, Schulich School of Medicine & Dentistry, Western University, Room B3-431A, 800 Commissioners Road East, London, ON N6A 5W9 Canada; 2 Department of Pathology & Laboratory Medicine, Schulich School of Medicine & Dentistry, Western University, London, ON Canada; 3 Princess Margaret Cancer Centre, University Health Network, Toronto, ON Canada; 4 Department of Oncology, Schulich School of Medicine & Dentistry, Western University, London, ON Canada; 5 Department of Microbiology and Immunology, Schulich School of Medicine & Dentistry, Western University, London, ON Canada; 6 Eli and Edythe Broad Center of Regenerative Medicine and Stem Cell Research, University of California, Los Angeles, CA USA; 7 Institute for Precision Health, University of California, Los Angeles, CA USA; 8 Jonsson Comprehensive Cancer Centre, University of California, Los Angeles, CA USA; 9 Department of Medical Biophysics, University of Toronto, Toronto, ON Canada

**Keywords:** Alpelisib, BYL719, PI3-kinase, PI3K, Head and neck cancer, HNSCC, AXL, TYRO3

## Abstract

**Background:**

Aberrant activation of the phosphatidylinositol 3-kinase (PI3K) pathway is common in many malignancies, including head and neck squamous cell carcinoma (HNSCC). Despite pre-clinical and clinical studies, outcomes from targeting the PI3K pathway have been underwhelming and the development of drug resistance poses a significant barrier to patient treatment. In the present study, we examined mechanisms of acquired resistance to the PI3Kα inhibitor alpelisib (formerly BYL719) in HNSCC cell lines and patient-derived xenografts (PDXs).

**Methods:**

Five unique PDX mouse models and three HNSCC cell lines were used. All cell lines and xenografts underwent genomic characterization prior to study. Serial drug treatment was conducted in vitro and in vivo to develop multiple, clinically-significant models of resistance to alpelisib. We then used reverse phase protein arrays (RPPAs) to profile the expression of proteins in parental and drug-resistant models. Top hits were validated by immunoblotting and immunohistochemistry. Flow cytometric analysis and RNA interference studies were then used to interrogate the molecular mechanisms underlying acquired drug resistance.

**Results:**

Prolonged treatment with alpelisib led to upregulation of TAM family receptor tyrosine kinases TYRO3 and AXL. Importantly, a significant shift in expression of both TYRO3 and AXL to the cell surface was detected in drug-resistant cells. Targeted knockdown of TYRO3 and AXL effectively re-sensitized resistant cells to PI3Kα inhibition. In vivo, resistance to alpelisib emerged following 20–35 days of treatment in all five PDX models. Elevated TYRO3 expression was detected in drug-resistant PDX tissues. Downstream of TYRO3 and AXL, we identified activation of intracellular MAPK signalling. Inhibition of MAPK signalling also re-sensitized drug-resistant cells to alpelisib.

**Conclusions:**

We have identified TYRO3 and AXL receptors to be key mediators of resistance to alpelisib, both in vitro and in vivo. Our findings suggest that pan-TAM inhibition is a promising avenue for combinatorial or second-line therapy alongside PI3Kα inhibition. These findings advance our understanding of the role TAM receptors play in modulating the response of HNSCC to PI3Kα inhibition and suggest a means to prevent, or at least delay, resistance to PI3Kα inhibition in order to improve outcomes for HNSCC patients.

## Background

Head and neck squamous cell carcinoma (HNSCC), which arises in the mucosa of the oral cavity, pharynx and larynx, is the 6th most common cancer worldwide [1]. Despite advances in available treatments (surgery, radiation, chemotherapy), survival rates at 5 years remain poor. Further, even patients cured by conventional treatment are frequently left with impairments in their abilities to speak, swallow and breathe, as well as facial disfigurements [2]. The development and clinical implementation of targeted therapeutics is needed to improve the survival outcomes and relieve the toxic burden associated with current HNSCC treatments.

The phosphatidylinositol 3-kinase (PI3K)/Akt/mTOR pathway is a major growth signalling pathway that regulates a variety of cellular processes, including protein and lipid synthesis, proliferation and cell survival [3]. The PI3K pathway is the most frequently dysregulated pathway in HNSCC, across both HPV-positive and HPV-negative HNSCC tumors [4–6]. Dysregulation of PI3K signalling—stemming from activating mutations or amplifications of *PIK3CA*—leads to constitutive activation of the pathway, which can promote tumor development and progression [5–7]. Given the prevalence of PI3K pathway alterations in HNSCC and the role this network plays in tumorigenesis, inhibiting this pathway is a logical therapeutic approach [7].

Various inhibitors that target one or more of the PI3K isoforms have entered clinical trials [7]. In fact, the α-isoform specific PI3K inhibitor alpelisib (Piqray®, Novartis) was recently approved by the US Food and Drug Administration (FDA) for treatment of *PIK3CA*-mutated, advanced or metastatic breast cancer. To date however, PI3K inhibitors as single agents have generally displayed limited efficacy. These drugs have typically led to cytostasis, rarely inducing tumor cell death or shrinkage [7, 8]. Moreover, in patients who initially respond to targeted PI3K inhibition, acquired resistance over time has been cited [9].

Acquired resistance to PI3K inhibition is an area of active research [9–14]. In ovarian cancer, elevated expression of receptor tyrosine kinases (RTKs), including HER2 and EGFR, as well as increased activation of Src, c-Jun and STAT3 have been implicated in mediating resistance to PI3K inhibition by NVP-BEZ235 [11]. In breast cancer, genetic alterations in *PTEN* resulting in loss of expression have been identified in a patient who initially achieved a clinical response to PI3K inhibition before progressing rapidly [9]. Only a limited number of studies to date have examined acquired resistance to PI3K inhibition in HNSCC. Of these, resistance to the pan-PI3K inhibitor BKM120 has been shown to involve positive feedback activation of IL-6/ERK signalling, while resistance to the α-isoform specific PI3K inhibitor alpelisib has been associated with growth signalling through the PLCγ-PKC network, downstream of the RTK AXL [12, 15]. It is evident that a number of distinct mechanisms and mediators of resistance to PI3K inhibition exist and may be context-specific according to the drug used and/or cancer type.

As mentioned, alpelisib (formerly BYL719) is an α-isoform specific PI3K inhibitor. It has been shown to exhibit “on-target” PI3K inhibition and anti-cancer efficacy, collectively leading to its recent FDA approval for breast cancer treatment [7, 8, 16]. Alpelisib targets the p110α catalytic subunit of the Class IA PI3K enzyme encoded by *PIK3CA* [17]. Due to the prevalence of genomic aberrations in *PIK3CA* observed in HNSCC, including gain of function mutations and amplifications, alpelisib is a particularly relevant drug. Further, by targeting only the α-isoform, alpelisib has shown to have better tolerability than other, broader-acting PI3K inhibitors, with generally manageable side effects (e.g. hyperglycemia) [8]. To date, there have been few investigations of how resistance to PI3K inhibition by alpelisib is acquired in the context of HNSCC [12]. Further, most studies have been limited to in vitro investigations and have not made use of patient-derived xenograft (PDX) models to explore resistance and/or validate their findings [12, 18].

To capitalize on the promise of PI3K inhibitors in HNSCC, it is essential to understand resistance mechanisms that may be acquired over time; this will enable the design of drug combinations that will be both tolerable and durable [19]. In the present study, we explored acquired resistance to alpelisib using both HNSCC cell lines and HNSCC PDXs. We observed elevated expression of the AXL RTK, in line with other studies, as well as elevation of its family member TYRO3 in alpelisib-resistant HNSCC models [12]. Further, we interrogated MAPK pathway activation downstream of AXL and TYRO3 as a critical network for circumventing PI3K inhibition. Collectively our findings emphasize TYRO3 and AXL as key mediators of acquired resistance to PI3K inhibition in HNSCC, through the MAPK pathway. Pan-TAM inhibition may be a promising second-line therapy for HNSCC patients receiving PI3K-targeted agents.

## Materials & methods

### Cell lines and chemical compounds

Cell lines were obtained from the sources listed (Additional Table 1). We previously used short tandem repeat profiling (The Center for Applied Genetics; Toronto) to confirm cell line identities [20]. 93-VU-147T cells were cultured in DMEM/F12, with 10% fetal bovine serum (FBS; GIBCO), penicillin (100 IU/mL; Invitrogen) and streptomycin (100 μg/mL; Invitrogen). Cal33 cells were cultured in DMEM, with 10% heat-inactivated FBS, 1x non-essential amino acids (Wisent), penicillin (100 IU/mL) and streptomycin (100 μg/mL). Resistant cell lines were obtained after chronic exposure to increasing concentrations of alpelisib for 3–4 months [22]. All cells were maintained in a 37 °C humidified atmosphere at 5% CO_2_. The inhibitors alpelisib and BI-D1870 were purchased from Selleckchem. Compounds were dissolved in DMSO for in vitro experiments.

### Establishment of patient derived xenografts

Mice were handled in accordance with the AUP 1542 approved by the University Health Network Animal Care Committee and in accordance with the CCAC regulations. Xenografts were established and handled as described previously [21]. Details are provided as Supplemental Methods & Materials.

Once tumor volumes reached 80-120 mm^3^ mice were randomized to either daily (5x/week) alpelisib (Novartis; 50 mg/kg) by oral gavage or a vehicle control (corn oil) [12, 21]. Individual tumor volumes were calculated using the formula: [length x (width)^2^] × 0.52. Where possible, STR profiling was used to confirm matching identities of primary tumors, xenograft tumors, patient blood and PDX-derived cell lines where available (Additional Table 2). Tumors were classified as HPV-positive using immunohistochemistry (IHC) for p16.

### Dose response curves

Cells were seeded in 96-well plates at 2400 cells/well and cultured overnight. Drugs were then added over 10-point ranges (0-40 μM). Viability was determined 72 h later using the PrestoBlue® Reagent (Thermo Fisher Scientific) on a Synergy™ H4 Hybrid Reader (BioTek) with 560 nm excitation and 590 nm emission wavelengths. For each dose, viability values were normalized to no-drug controls and average viability for each dose was calculated. To determine the half-maximal inhibitory concentration (IC_50_) values, normalized relative fluorescence values of drug-treated replicates were calculated as a percentage of the mean RFU of the control replicates and then drug doses were transformed to a logarithmic scale. IC_50_ values were subsequently calculated by non-linear regression. Values are plotted as mean + standard deviation (SD) using Prism® 7 GraphPad Software.

### Clonogenic survival assay

Parental and resistant cell lines were counted and seeded at 500 cells per well into 24-well dishes. Cells were allowed to adhere for 48 h and then were treated with media containing 5 μM alpelisib. For the next 7–14 days, cells were monitored and media replaced every 3 days until visible colonies were formed. Colonies were rinsed with 1x PBS, fixed with cold 100% methanol (MeOH) and stained with 0.5% crystal violet in 25% MeOH/1x PBS. The colonies were then gently washed with water and air-dried. Visible colonies were counted.

### Reverse phase protein arrays

Cells were prepared for reverse phase protein arrays (RPPAs) as follows: 10 cm plates were washed twice with cold 1x PBS. Cold lysis buffer (containing: 1% Triton X-100, 50 mM HEPES pH 7.4, 150 mM NaCl, 1.5 mM MgCl2, 1 mM EGTA, 100 mM NaF, 10 mM Na pyrophosphate, 1 mM Na3VO4, 10% glycerol and 1% freshly-added protease and phosphatase inhibitors) was added to the plates which were then incubated 20mins on ice with occasional shaking. Lysed cells were centrifuged at 14000 rpm for 10mins at 4 °C. Protein concentration determined by Bradford Assay. Lysates were combined with sample buffer (40% glycerol, 8% SDS, 0.25 M Tris-HCl pH 6.8 and 1/10 volume β-mercaptoethanol –added just before use) at 3 parts lysate:1 part sample buffer. Samples were boiled for 5 mins and stored at − 80 °C.

Samples were submitted to MD Anderson’s Functional Proteomics RPPA Core Facility. Briefly, lysates were serially diluted and arrayed onto nitrocellulose-coated glass slides. Samples were probed with 307 antibodies and visualized by DAB colorimetric reaction. Slides were then scanned and spot densities quantified by Array-Pro Analyzer. All data points were normalized for protein loading and transformed to a linear value. Resistant replicates were then normalized to the mean of their respective parental replicates. Values were then log2-transformed and we restricted our analysis to the top 50% of differentially-expressed proteins for each cell line. Unsupervised hierarchical clustering was performed using the average agglomeration method and Euclidean distance measurements. Clustering was performed in R using the ComplexHeatmap package (version 2.1.1).

### Immunoblotting and densitometric analysis

Cell lysates were prepared for immunoblotting as described previously [23]. A list of primary antibodies used is provided in Additional Table 3. Membranes were visualized following exposure to enhanced chemiluminescence reagent (Luminata™ Crescendo or Luminata™ Forte, Western HRP Substrate; Millipore) on a Bio-Rad ChemiDoc™MP Imaging System.

ImageJ was used to select and determine the background-subtracted density of the bands in all immunoblots. Values were then normalized to their corresponding α-tubulin band. All values are presented below the associated band.

### Tissue microarray (TMA) and immunohistochemistry

TMAs were constructed for two of the xenograft models. In brief, the FFPE block for each tumor was sectioned and stained with hematoxylin & eosin (H & E) to confirm the presence of human tumor. Guided by these sections, a Manual Tissue Arrayer (MTA-1; Beecher Instruments Inc.) was used to punch out 3–4 cylindrical cores of 0.6 mm diameter from each sample. Cores were arrayed into recipient paraffin blocks. Eleven control tissues (tonsil, stomach, prostate, pancreas, lung, kidney, skin, thyroid, spleen, adipose, liver) were also included on each block. Cores were sealed into recipient blocks by heating at 40 °C for ~40mins. Blocks were sectioned into 1.5 μM sections and affixed to glass slides. Every ninth slide was stained with H & E to provide a reference. Additional details are available in the MTA-1 Instruction Manual (www.beecherinstruments.com). IHC staining was completed in collaboration with the Department of Pathology & Laboratory Medicine and the Molecular Pathology Core Facility (Western University). Tissues were examined using an Aperio ScanScope® slide scanner and staining quantification was performed using the Fiji plugin for ImageJ.

### Flow cytometry for cell surface expression of RTKs

Parental and resistant cells were collected by trypsinization, washed in 1x PBS and counted. Single-cell suspensions were incubated in a 5% BSA solution containing anti-AXL or TYRO3, PE-conjugated antibodies at 1:50 (R & D Biosystems) for 40mins in the dark at room temperature. Cells were passed through a cell strainer to collect single cells and were protected from light until they were quantified using a Beckman-Coulter Cytomics FC500 flow cytometer with at least 10,000 events counted per test. Histograms were used to compare intensity of staining between unstained, parental and resistant cell line samples. Median fluorescence intensity was calculated for each sample and *t-*tests were used to quantify differences.

### RNA interference

Knockdown of AXL and TYRO3 was performed using specific pooled siRNAs purchased from Dharmacon (Cat No’s. L-003104-00-0005 and L-003183-00-0005, respectively), as described previously [23]. Scrambled control siRNA (siCT) (Thermo Fisher Scientific; Cat No. 4390843) was also used. Knockdowns were confirmed by immunoblotting.

For drug testing, cells were seeded into 96-well dishes at 2400cells/well. Alpelisib was added the next day at 5 μM and cells were incubated for 72 h. Cell viability was then determined indirectly using the PrestoBlue® Reagent (Thermo Fisher Scientific) on a Synergy™ H4 Hybrid Reader (BioTek) with 560 nm excitation and 590 nm emission wavelengths. For each condition, alpelisib-treated cells were compared with normalized untreated cells to determine the relative effect of RNAi-mediated knockdown.

### Generation of PDX-derived cell line

Using cells dissociated from first-passage xenograft tumors, we attempted to establish cell lines from the patient tumors that were used to generate the PDX models (Additional Fig. 1a). Specifically, the generation of cell lines was attempted from tumor tissues that were never treated with alpelisib nor the vehicle agent. A cell line (called PDX-C Cell Line) was successfully established from one model, PDX-C (Additional Fig. 1b). STR profiling, immunoblotting and flow cytometry for cell surface expression of EpCAM (CD326) were all completed as described previously, validating the line as a human epithelial line from the same patient as the PDX-C tumor (Additional Fig. 1c, Additional Table 2).

### Statistical analysis

All analyses were performed with Prism® 7 GraphPad Software. Experimental groups were compared with controls using Student’s unpaired, two-tailed *t-*tests. Multiple groups were compared across a single condition using one-way ANOVA. *P* < 0.05 was used to define significant differences from the null hypothesis.

## Results

### Alpelisib inhibits growth and PI3K signalling in HNSCC cells

**Fig. 1:**
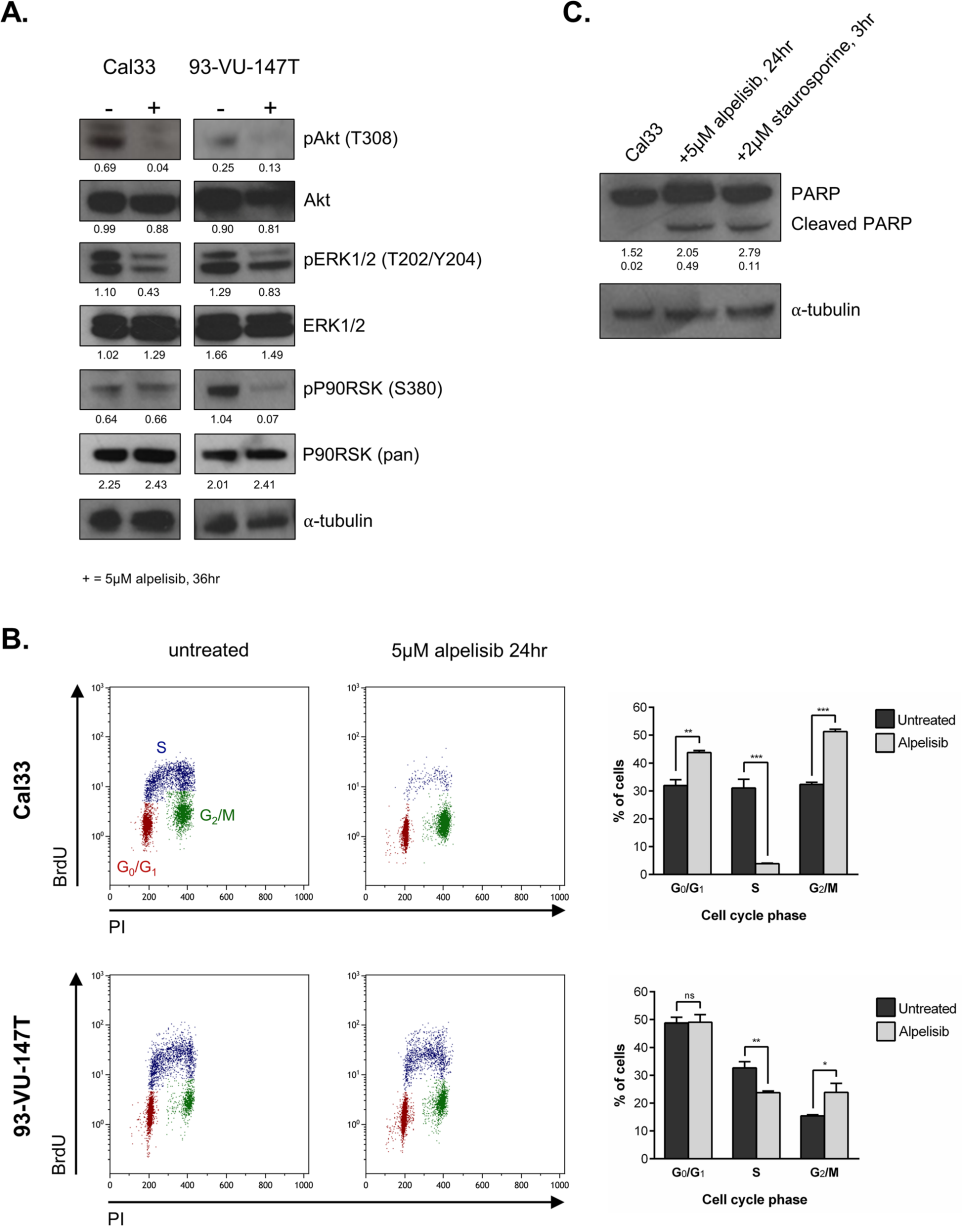
Alpelisib inhibits growth and PI3K signalling in HNSCC cells. **a** Immunoblot of PI3K and MAPK pathway members Akt, ERK1/2 and P90RSK following treatment with 5 μM alpelisib for 36 h. Densitometric quantification of each protein relative to α-tubulin is shown below each band. **b** Flow cytometric analysis of Cal33 and 93-VU-147T cells treated with alpelisib (5 μM) for 24 h (3 replicates per line) before BrdU incorporation and labeling with propidium iodide. Approximately 10,000 events were counted per test (5000 events are shown in each figure). Proportion of cells in each cell cycle phase is shown, + standard deviation. * represents *p* < 0.05, ** represents *p* < 0.01, *** represents *p* < 0.001, ns = not significant, unpaired Student’s *t*-test. **c** Immunoblot for PARP cleavage in Cal33 cells treated with alpelisib (5 μM) for 24 h, or staurosporine (2 μM) for 3 h, as a positive control. Densitometric quantification of each protein relative to α-tubulin is shown below each band

Prior to exploring resistance to PI3K inhibition, we first validated the efficacy of alpelisib in HNSCC cell lines. Alpelisib treatment reduced signalling through both the PI3K and MAPK pathways in Cal33 (*PIK3CA* mutant) cells and 93-VU-147T (*PIK3CA* amplified) cells (Fig. 1a), as indicated by reduced levels of phosphorylated (p)-Akt (Thr308), pERK1/2 (Thr202/Tyr204) and pP90RSK (Ser380) [21]. Based on previous studies, we hypothesized that the mechanism of action for alpelisib in HNSCC would involve cell cycle arrest [7]. Following 24 h of treatment with alpelisib, we observed a significant reduction in the proportion of proliferating (S-phase) cells (Fig. 1b) [24, 25]. To determine whether alpelisib was also able to induce cell death through apoptosis, we examined PARP cleavage (Fig. 1c). Following alpelisib treatment, cleaved PARP was readily detectable. Of note, we have also previously characterized the sensitivity of 28 HNSCC cell lines to PI3K inhibition and found both wildtype and *PIK3CA*-altered cell lines to be sensitive to PI3K inhibition [21].

### Genomically-distinct HNSCC cell lines develop resistance to alpelisib

**Fig. 2:**
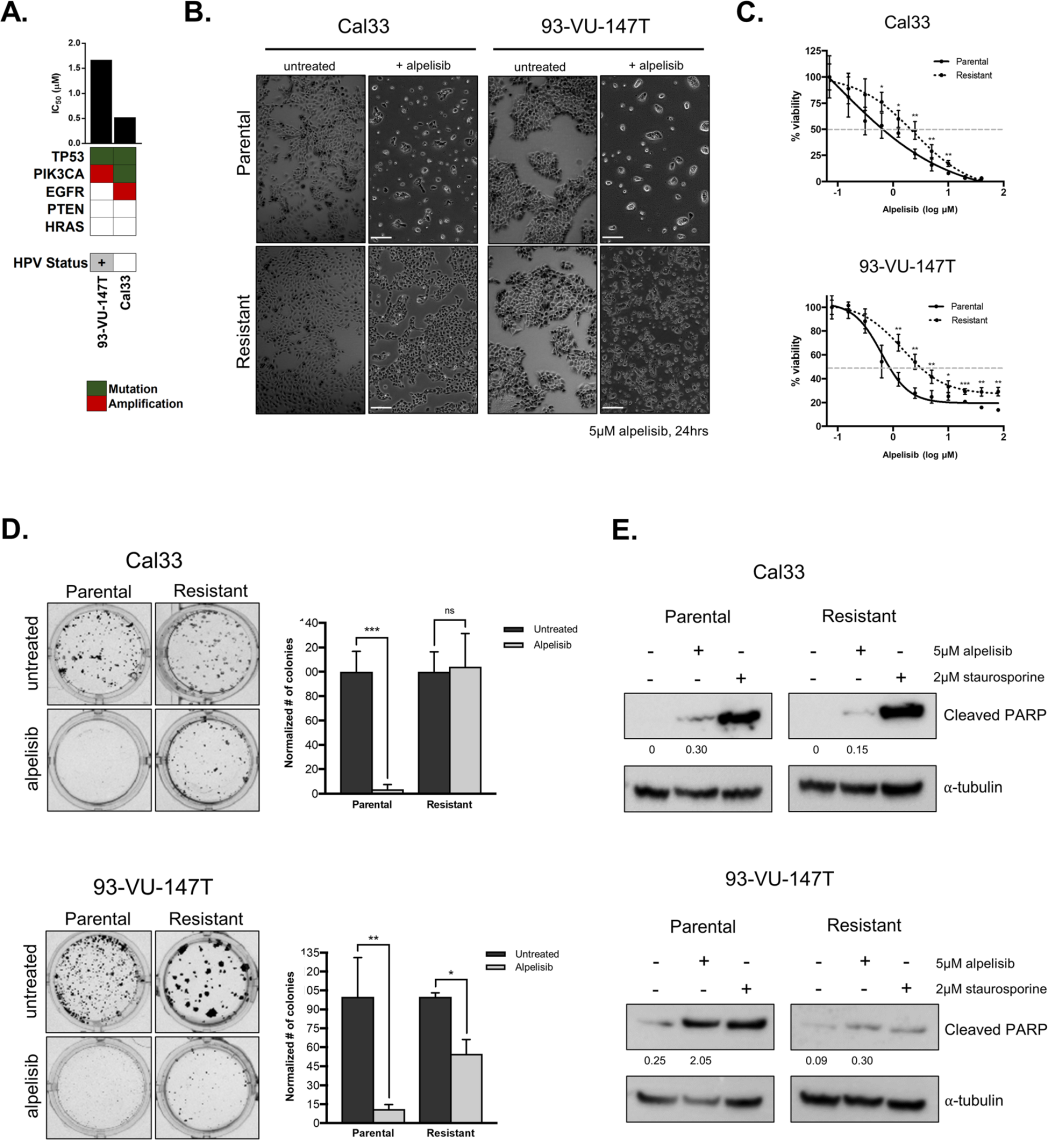
Genomically-distinct HNSCC cell lines become resistant to PI3K inhibition over time. **a** Genomic features and IC_50_ values for Cal33 and 93-VU-147T cell lines. **b** Phase contrast microscopy images of parental and resistant HNSCC cell lines, with and without 5 μM alpelisib treatment for 24 h. **c** Dose response curves comparing sensitivity of parental and resistant cell lines over 10 doses of alpelisib. **d** Colony formation assays comparing tolerance of parental and alpelisib-resistant cell lines to alpelisib treatment over time. Number of colonies was counted and graphed. * represents *p* < 0.05, ** represents *p* < 0.01, *** represents *p* < 0.001, ns = not significant, unpaired Student’s *t*-test. **e** Immunoblot for PARP cleavage in Cal33 and 93-VU-147T parental and alpelisib-resistant cells treated with alpelisib (5 μM) for 45 h, or staurosporine (2 μM) for 3 h, as a positive control. Densitometric quantification of each protein relative to α-tubulin is shown below each band

To identify pathways associated with acquired resistance to alpelisib, we exposed two genomically-distinct HNSCC cell lines (Fig. 2a) to increasing concentrations of alpelisib over a 3–4 month period (schematic shown in Additional Fig. 2), ultimately yielding cell lines significantly more resistant to alpelisib than their parental counterparts (Fig. 2b-c) [21]. To verify the durability of the resistant cell lines, parental and resistant cells were challenged to grow as single cell colonies in the presence of alpelisib. Whereas alpelisib treatment led to a significant reduction in the number of colonies formed by both parental cell lines, it was much less effective in resistant cell lines (Fig. 2d). Although the alpelisib-resistant 93-VU-147T cells exhibited a modest reduction in colony formation following alpelisib treatment, the difference was much less than that of the parental line. Thus, cell lines treated for a prolonged period with alpelisib exhibit increased tolerance for this PI3K inhibitor. In addition, we examined the relative abundance of colony sizes (small, medium, large) in parental versus resistant cell lines as this reflects cell lines’ proliferative status (Additional Fig. 3a-b). Finally, to compare the ability of alpelisib to induce cell death through apoptosis, we examined PARP cleavage following alpelisib treatment in parental and alpelisib-resistant cell lines (Fig. 2e). We found that following alpelisib treatment, cleaved PARP was readily detectable in parental cells, but was less abundant in alpelisib-resistant cells, suggesting that alpelisib treatment is less effective at inducing cell death via apoptosis in cells that have acquired resistance to alpelisib.

**Fig. 3:**
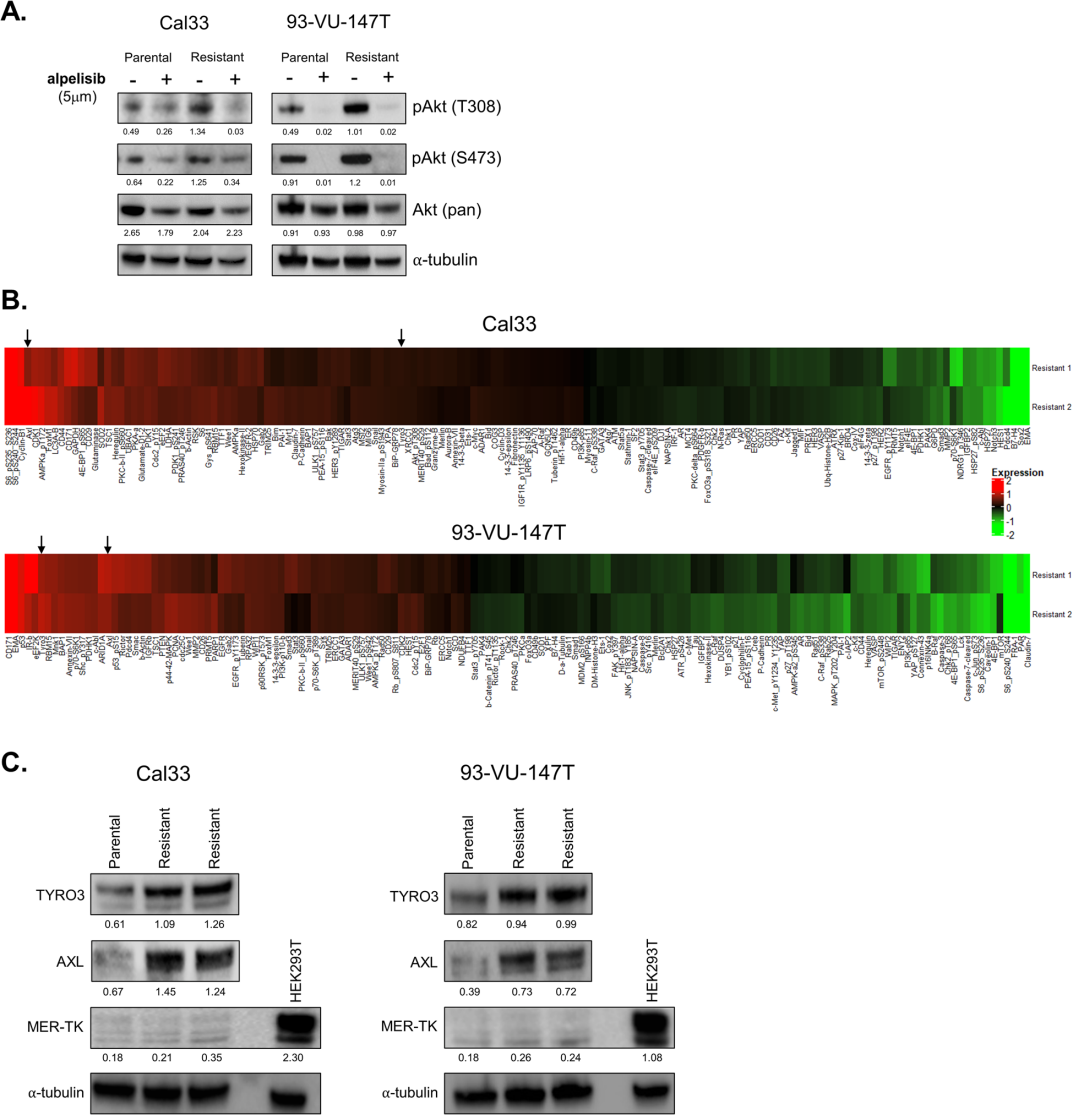
Expression of AXL and TYRO3 is elevated in alpelisib-resistant cells. **a** Immunoblot with/without 5 μM alpelisib (36 h) in parental and resistant Cal33 and 93-VU-147T cell lines using indicated antibodies. Densitometric quantification of each protein relative to α-tubulin is shown below each band. **b** Heatmaps displaying RPPA results (top 50% of differentially-expressed proteins for each cell line is shown). Arrows indicate AXL and TYRO3. **c** Immunoblot showing expression of TAM family RTKs AXL, TYRO3 and MER-TK in parental and alpelisib-resistant HNSCC cell lines. HEK293T cells are included as a positive control for MER-TK expression. Densitometric quantification of each protein relative to α-tubulin is shown below each band

To determine whether alpelisib continued to block signalling through the PI3K pathway in drug-resistant cells, we used immunoblotting to examine Akt phosphorylation following alpelisib treatment in parental and resistant cell lines. In parental and drug-resistant cell lines alike, alpelisib treatment suppressed Akt Thr308 and Ser473 phosphorylation, indicating that resistance was acquired via a compensatory mechanism and not loss of direct inhibition of PI3K activity (Fig. 3a).

### Expression of AXL and TYRO3 is elevated in alpelisib-resistant cells

To examine a broad array of signalling pathways that could mediate resistance to PI3K inhibition in HNSCC cells, we performed RPPAs on lysates from Cal33 and 93-VU-147T parental cells and resistant cell lines (Fig. 3b). In both resistant cell lines, RPPAs suggested that expression of the membrane-bound RTK AXL was elevated relative to parental cells. As mentioned, AXL has been previously shown to mediate resistance to various anti-cancer agents, including EGFR, HER2 and PI3K-targeted therapies [12, 26–28]. AXL is part of a three-member RTK sub-family known as the ‘TAM family’ of receptors (**T**YRO3, **A**XL, **M**ER-TK) [29–32]. Interestingly, expression of TYRO3 was also found to be elevated in both Cal33 and 93-VU-147T alpelisib-resistant cells. To our knowledge, TYRO3 has never been implicated in PI3K-inhibitor resistance, nor in HNSCC as an effector of therapy response. In general, much less is known about TYRO3, including the role it plays in cancer development and progression [31–34].

We confirmed elevated protein expression of AXL and TYRO3 in alpelisib-resistant Cal33 and 93-VU-147T cells, relative to parental cells by immunoblotting (Fig. 3c). We also examined expression of the third member of the TAM RTK family, MER-TK, which was not included in the RPPA. MER-TK was weakly detectable in both HNSCC cell lines, in contrast to its high expression in HEK293T cells (long exposure blots shown in Additional Fig. 4a-b). As MER-TK was only weakly detected in both cell lines and no difference in expression was apparent between parental and drug-resistant cells, we did not examine it further. Based on our findings, we hypothesized that overexpression of TYRO3 and/or AXL receptors could play a role in mediating resistance to PI3Kα inhibition.

### HNSCC PDX models develop resistance to alpelisib

**Fig. 4:**
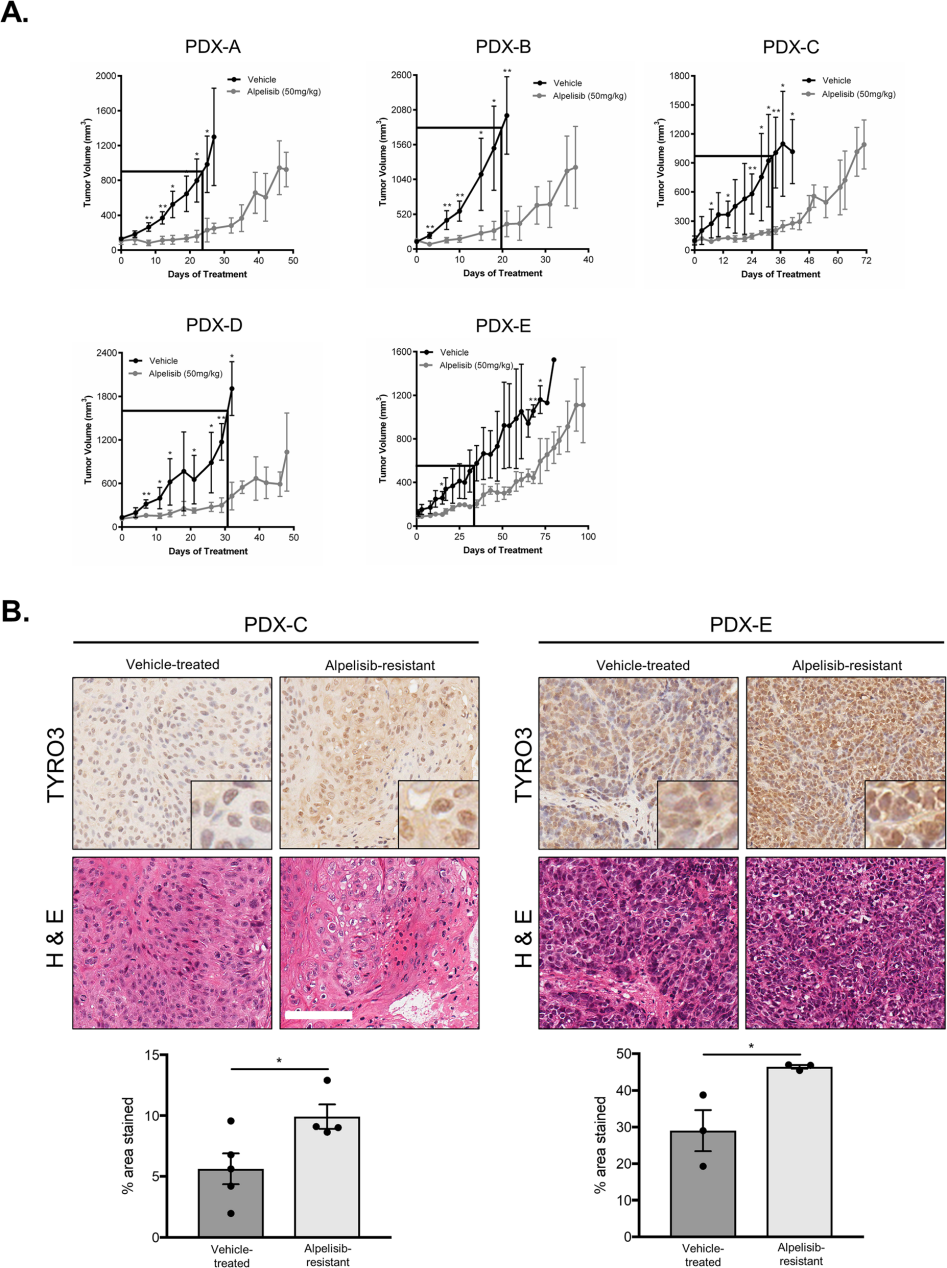
Genomically-distinct HNSCC PDX models develop resistance to alpelisib following prolonged treatment. **a** Growth curves for PDX models treated over time with alpelisib. 5 mice per arm received either alpelisib (50 mg/kg) or a vehicle agent (corn oil). * represents *p* < 0.05, ** represents *p* < 0.01, unpaired Student’s *t*-test. Boxed out region highlights early treatment days where alpelisib-treated tumors showed static growth relative to the vehicle treatment. **b** Representative IHC sections showing TYRO3 staining in PDX-C and PDX-E models. Quantification was completed using Fiji software and is shown below. * represents *p* < 0.05, unpaired Student’s *t*-test. Scale bars represent 100 μM

In parallel with our cell line models, we generated 5 PDX models from HNSCC patient tumors (clinical characteristics outlined in Additional Table 4). Of the 5 models, one (PDX-B) was both HPV-positive and contained a non-synonymous *PIK3CA* mutation (E545K) [21]. The remaining models were all *PIK3CA* wildtype. Several of the PDX models contained mutations frequently seen in HNSCC, including *TP53* mutations, *EGFR* amplifications and *CDKN2A* alterations [4, 21]. Histological comparison of PDXs and their corresponding primary tumors (where available) revealed a high degree of similarity in their cellular morphology (Additional Fig. 5). In all 5 PDX models, treatment with alpelisib significantly suppressed tumor growth for the first 20–35 days, relative to the vehicle agent (Fig. 4a, boxed regions). However, beyond this initial response period, alpelisib-treated tumors began to resume growth or exhibit an increased rate of growth over time (Fig. 4a). Thus, PDX models behave similarly to cell lines in that they also spontaneously develop resistance to PI3Kα inhibition by alpelisib over time.

Ki67 staining was used to examine the proliferative activity of vehicle-treated tumors, alpelisib-sensitive tumors (not treated to resistance) and alpelisib-resistant tumors. Most vehicle-treated and alpelisib-resistant tumors exhibited strong positive Ki67 staining, whereas alpelisib-sensitive tumors showed weaker staining (representative sections shown in Additional Fig. 6). We next analyzed the expression of AXL and TYRO3 in PDX models using IHC. While no difference in AXL expression was detected for either model examined (Additional Fig. 7), TYRO3 expression following prolonged treatment with alpelisib was elevated in both PDX models (Fig. 4b).

### TYRO3 and AXL overexpression mediates resistance to alpelisib

**Fig. 5:**
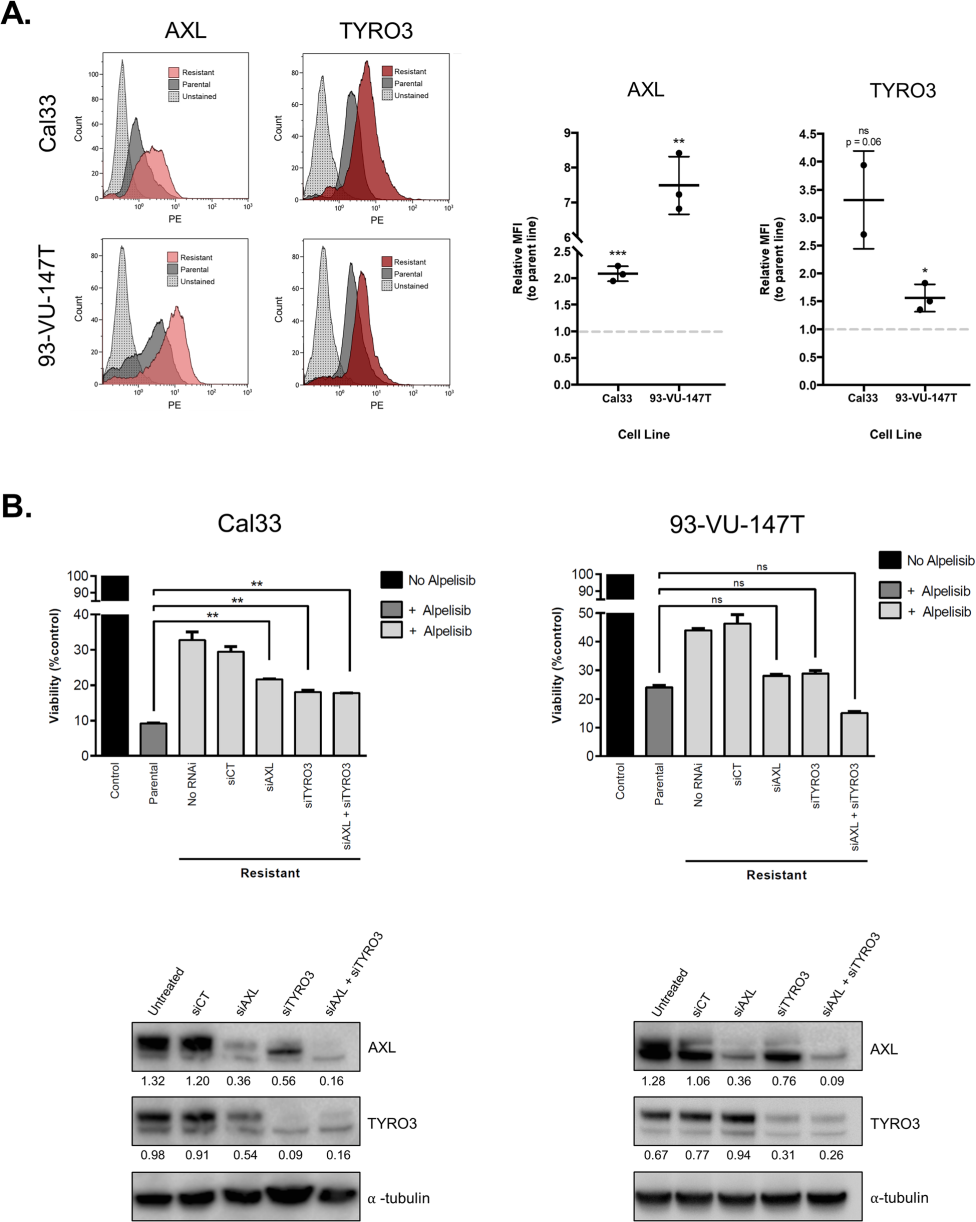
TYRO3 and AXL modulate sensitivity to alpelisib. **a** Flow cytometric analysis of AXL and TYRO3 in parental and resistant HNSCC cell lines. Median fluorescence intensity (MFI) was measured and graphed for three biological replicates. * represents *p* < 0.05, ** represents *p* < 0.01, *** represents *p* < 0.001, **** represents *p* < 0.0001, ns = not significant, unpaired Student’s *t*-test. **b** siRNA-mediated knockdown of AXL (siAXL) and TYRO3 (siTYRO3) in Cal33 and 93-VU-147T cells. siCT = scrambled control siRNA, RNAi = RNA interference. ** represents *p* < 0.01, ns = not significant. One-way ANOVA. Immunoblot of AXL and TYRO3 expression following siRNA-mediated knockdowns is shown below, siCT = scrambled control siRNA. Densitometric quantification of each protein relative to α-tubulin is shown below each band

Since expression of AXL and TYRO3 were both elevated in alpelisib-resistant models, we proceeded to determine the relative expression of both receptors at the cell surface in parental and resistant cells. Flow cytometric analysis demonstrated a significant increase in both AXL and TYRO3 surface levels in alpelisib-resistant Cal33 and 93-VU-147T cells, compared to parental cells (Fig. 5a).

To assess whether the upregulation of TYRO3 and/or AXL expression plays a causative role in mediating resistance to PI3Kα inhibition, we used siRNAs to silence each receptor in alpelisib-resistant cells. Knockdown of either TYRO3 or AXL re-sensitized resistant cells to alpelisib treatment, to a level almost comparable to their parental (baseline) sensitivity (Fig. 5b). In 93-VU-147T cells, combined TYRO3 and AXL knockdown sensitized resistant cells to an even greater extent than their parental sensitivity, highlighting the role both receptors play in modulating response to alpelisib treatment.

### MAPK signalling is activated in alpelisib-resistant models

**Fig. 6:**
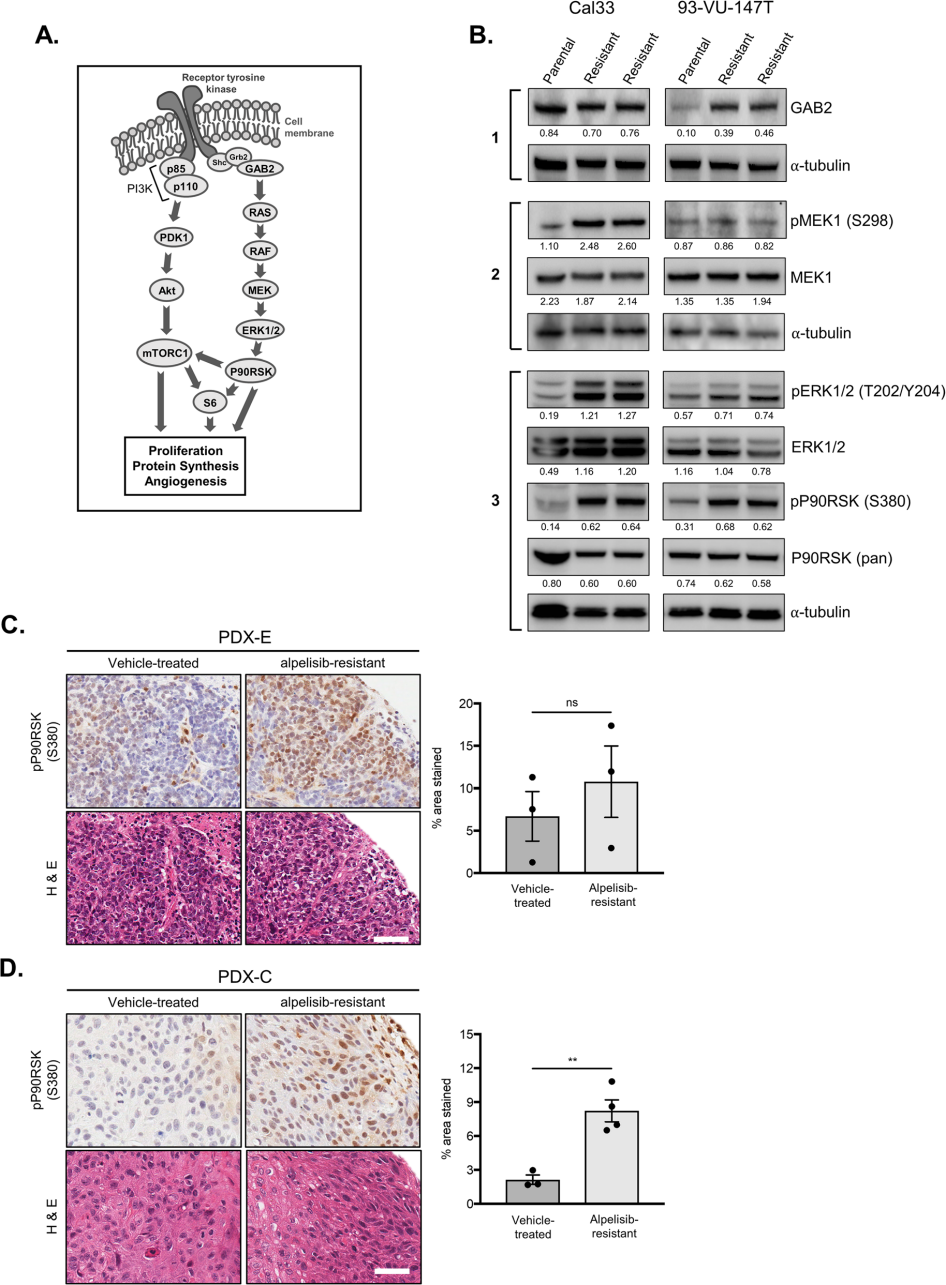
Activation of the MAPK signalling pathway in alpelisib-resistant cell lines and PDX models. **a** Schematic representation of PI3K and MAPK pathways, with crosstalk activating mTORC1 shown. **b** Immunoblot with indicated parental and alpelisib-resistant lysates examining activation of the MAPK pathway. Densitometric quantification of each protein relative to α-tubulin is shown below each band. **c** & **d** Representative IHC sections showing pP90RSK (Ser380) staining in PDX-E **c** and PDX-C **d** models. Quantification was completed using Fiji software and is shown below. *** represent *p* < 0.01, ns = not significant, unpaired Student’s *t*-test. Scale bars represent 50 μM

Since the expression of activated MAPK pathway members ERK1/2 and P90RSK was reduced by PI3K inhibition in parental cells (Fig. 1a) and the MAPK signalling pathway is a downstream target associated with TAM RTKs, we proceeded to examine the activation status of several MAPK pathway members in our resistant cells (Fig. 6a) [33]. Beginning upstream, we examined expression of the scaffold protein GAB2 that mediates signalling from the adaptor protein Grb2 on intracellular RTK domains to RAS. GAB2 expression was elevated in alpelisib-resistant 93-VU-147T cells (as suggested in the RPPA), although no difference was apparent in Cal33 cells (Fig. 6b, block 1). The next differentially-expressed pathway member was phosphorylated MEK1 (Ser298) which was apparent in alpelisib-resistant Cal33 cells (Fig. 6b, block 2). Moving down the MAPK pathway, elevated activating phosphorylation of ERK1/2 and P90RSK was detected in all alpelisib-resistant cell lines (Fig. 6b, block 3). Collectively, these observations reveal an induction of MAPK pathway activation upon prolonged treatment with alpelisib.

Given the upregulation of pP90RSK (Ser380) observed in the resistant cell lines and its described involvement in HNSCC oncogenesis, we proceeded to examine its expression in our PDX models using IHC [35, 36]. We observed a non-significant trend towards elevated pP90RSK (Ser380) expression in alpelisib-resistant PDX-E tissues and a significant increase in expression in PDX-C tissues (Fig. 6c-d).

### Inhibition of MAPK signalling improves response to alpelisib

**Fig. 7:**
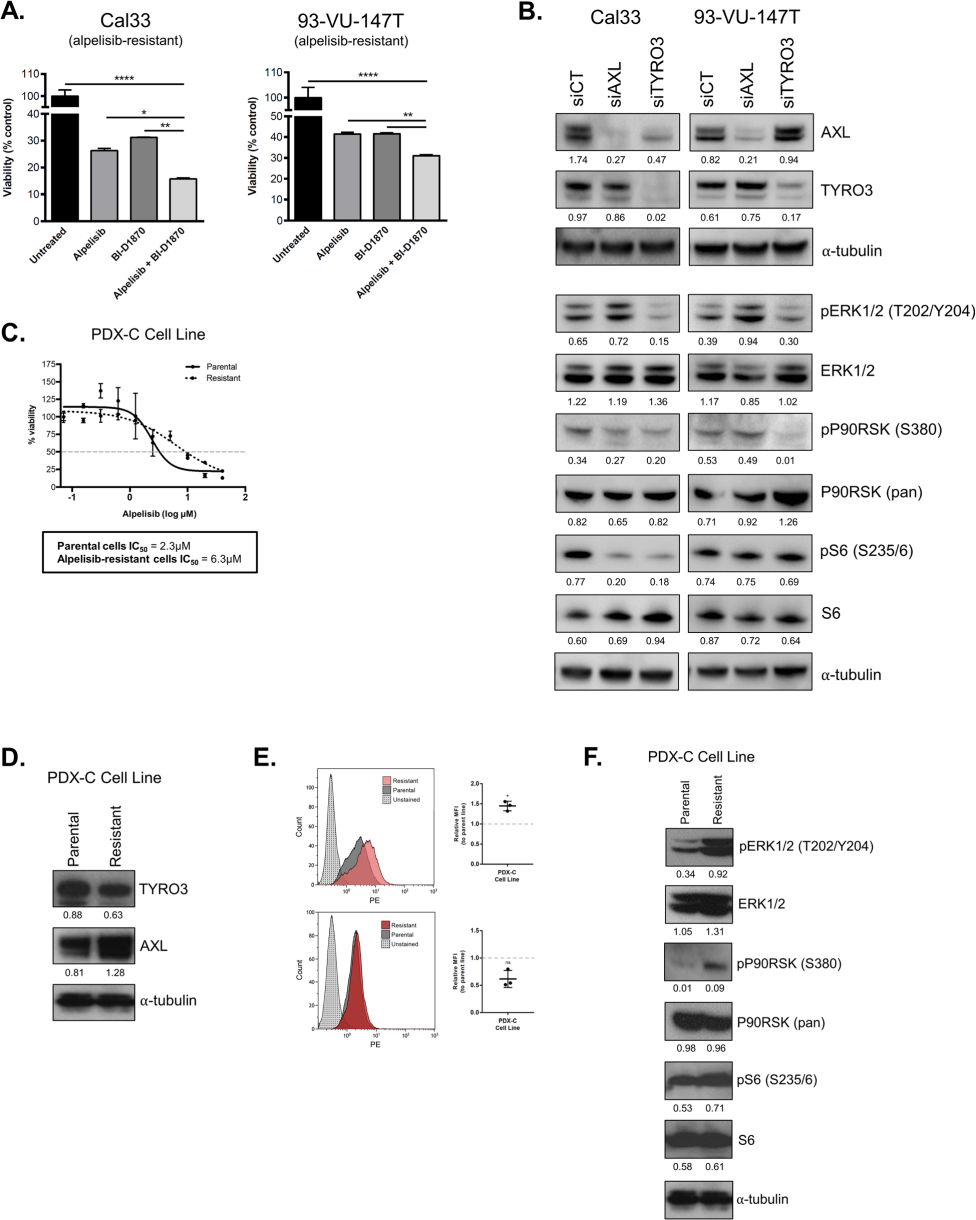
Inhibition of MAPK signalling improves response to alpelisib. **a** Effect of P90RSK inhibitor BI-D1870 and alpelisib (5 μM each) on viability of alpelisib-resistant cell lines. * represents *p* < 0.05, ** represents *p* < 0.01, **** represents *p* < 0.0001. One-way ANOVA. **b** Immunoblot of MAPK pathway members following knockdown of AXL and TYRO3. siCT = scrambled control siRNA. Densitometric quantification of each protein relative to α-tubulin is shown below each band. **c** Dose response curve comparing sensitivity of parental and alpelisib-resistant PDX-C cells to alpelisib. **d** Immunoblot of parental and alpelisib-resistant PDX-C cell lysates for expression of TYRO3 and AXL RTKs. Densitometric quantification of each protein relative to α-tubulin is shown below each band. **e** Flow cytometric analysis of AXL and TYRO3 in parental and resistant PDX-C cells. Median fluorescence intensity (MFI) was measured and graphed for three biological replicates. * represents *p* < 0.05, ns = not significant, unpaired Student’s *t*-test. **f** Immunoblot of parental and alpelisib-resistant PDX-C cell lysates for activation of the MAPK pathway. Densitometric quantification of each protein relative to α-tubulin is shown below each band

To evaluate the effect of MAPK signalling on resistant cells’ responsiveness to alpelisib, we targeted the downstream MAPK pathway member P90RSK using the small molecule inhibitor BI-D1870, alone and in combination with alpelisib [37]. In both alpelisib-resistant cell lines, BI-D1870 treatment resulted in a significant reduction in cell viability (Fig. 7a). When BI-D1870 and alpelisib treatments were combined, greater decreases in cell viability were observed.

### Knockdown of TYRO3 and AXL reduces MAPK pathway activation

We next evaluated the relation between expression of TYRO3 and AXL, and MAPK pathway activation. Following knockdown of TYRO3 and AXL in alpelisib-resistant cells, we used immunoblotting to detect phosphorylated (active form) members of the MAPK pathway, including ERK1/2, P90RSK and S6 (Fig. 7b). In Cal33 cells, silencing of either TYRO3 or AXL was associated with reduced phosphorylation of P90RSK (Ser380) and S6 (Ser235/6), while only TYRO3 silencing reduced ERK1/2 (Thr202/Tyr204) phosphorylation. In 93-VU-147T cells, TYRO3 knockdown reduced phosphorylation of ERK1/2 and P90RSK, while AXL knockdown did not have an apparent effect on MAPK pathway activation.

### Baseline expression of AXL and TYRO3 is not associated with sensitivity to PI3K inhibition

To determine whether expression of TYRO3 and/or AXL was associated with PI3K inhibitor sensitivity at baseline (without prolonged drug exposure), we examined the protein expression of both receptors in a panel of 25 HNSCC cell lines (Additional Table 1) [20]. We previously characterized the sensitivity of all 25 lines to alpelisib and ordered the cell lines accordingly [21]. While expression of both proteins varied between cell lines, we did not observe a trend in the expression of either TYRO3 or AXL that appeared to correlate with sensitivity to alpelisib (Additional Fig. 8). Given an absence of a correlation between baseline TAM RTK expression and response to PI3K inhibition, it appears the involvement of TYRO3 and AXL in drug response is contingent on either the activity of the receptors or a relative upregulation of receptors during the course of treatment that subsequently affects drug response.

### Activation of MAPK signalling and elevated TAM RTK expression in a PDX-derived cell line

Finally, in parallel with our in vitro and in vivo models of acquired resistance to PI3K inhibition, we treated our PDX-derived cell line (PDX-C cell line) with alpelisib to generate an alpelisib-resistant cell line. This model system provided the unique opportunity to use an early-passage tumor-derived cell line to validate the data from both our in vitro studies using established HNSCC cell lines and our in vivo studies using PDX models. The alpelisib-resistant PDX-C-derived cell line had a ~ 3-fold increase in IC50 (2.3 μM versus 6.3 μM), relative to its parental counterpart (Fig. 7c). Immunoblotting revealed elevated expression of AXL in the alpelisib-resistant cell line, while TYRO3 expression appeared stable (Fig. 7d). We proceeded to evaluate the relative expression of AXL and TYRO3 at the cell surface using flow cytometry. We observed a significant increase in AXL surface levels in alpelisib-resistant PDX-C cells, compared to in parental cells (Fig. 7e). Downstream, elevated phosphorylation of MAPK pathway members ERK1/2 and P90RSK was also detected in alpelisib-resistant PDX-C cells, confirming our previous in vitro and in vivo PDX findings (Fig. 7f).

## Discussion

In this study, we demonstrate that PI3Kα inhibition exhibits anti-tumor efficacy in HNSCC models by dampening PI3K signalling, inducing PARP cleavage and reducing the proportion of actively-proliferating cells. As targeted PI3Kα inhibition is under active clinical investigation for HNSCC patients and has been FDA-approved for breast cancer treatment already, we proceeded to evaluate the efficacy of PI3Kα inhibition over time. We show in both in vitro and in vivo assays that HNSCC escapes the anti-tumor activity of alpelisib over a period of weeks to months. This acquisition of drug resistance is associated with upregulation of the RTKs TYRO3 and AXL, and an increase in signalling through the MAPK network. While AXL has been described in various settings to function as a mediator of acquired drug resistance, the involvement of its family member TYRO3 is previously unrecognized [12, 26, 27, 38, 39].

AXL and TYRO3 are two members of the three-membered TAM family of RTKs, which also includes MER-TK [32]. Although none of the TAM RTKs are considered to be strong oncogenes, all three have demonstrated transforming potential and it is increasingly recognized that their overexpression contributes to resistance to both standard and targeted chemotherapies [31, 40]. AXL is by far the best-studied TAM RTK and has an established role in supporting tumorigenesis through its positive effects on cellular survival, migration, proliferation and invasion, and in mediating acquired resistance [31]. To date, overexpression of AXL has been implicated in resistance to imatinib (BCR-Abl, c-Kit and PDGFR inhibitor), lapatinib (HER2 inhibitor), erlotinib (EGFR inhibitor) and cetuximab (EGFR-targeting monoclonal antibody), as well as resistance to the chemotherapeutics doxorubicin, cisplatin and etoposide (VP-16) in a variety of solid tumor types and blood cancers [26, 27, 31, 38, 39, 41, 42]. In contrast, TYRO3 overexpression has been shown to mediate taxol resistance in ovarian cancer and in general, promote progression of various cancers when overexpressed [31, 34, 43, 44].

In our HNSCC models, upregulation of both AXL and TYRO3 total protein was detected, as was an increase in cell surface localization in alpelisib-resistant versus parental samples. The involvement of AXL and TYRO3 in resistance to PI3Kα inhibition is underscored by the fact that knockdown of either or both receptors significantly sensitized cells to alpelisib treatment.

Across a large panel of HNSCC cell lines, we did not observe a trend between protein expression of TYRO3 or AXL, and sensitivity to PI3Kα inhibition. This leads us to believe that the involvement of AXL and TYRO3 in PI3K inhibitor resistance is likely based on a relative increase in expression/surface localization or altered receptor activity, rather than a baseline expression level. At present it is not well known how expression of AXL and TYRO3 is regulated; given the emerging role of TAM RTKs in cancer and drug response however, this is an area of active research [32, 33]. Hypoxia and HIF-1α expression has been associated with AXL expression while certain microRNAs (miRNAs) are also thought to be a mediator of TAM RTK expression [45–48].

Downstream of AXL and TYRO3, numerous intracellular signalling pathways have been associated with cancer progression and drug resistance [32, 33]. Re-activation of Akt signalling and activation of the NF-κB pathway are two such examples [38, 39]. In the context of HNSCC specifically, PLCγ-PKC signalling downstream of AXL has been identified following PI3Kα inhibition [12]. Our data provide evidence of MAPK pathway activation, consistent across both cell lines surveyed. Further, targeted inhibition of the downstream MAPK pathway member P90RSK, alone and in combination with alpelisib, resulted in a significant reduction in cell viability of alpelisib-resistant HNSCC cells, emphasizing the relevance of this pathway in circumventing PI3K inhibition. Our observations are in accordance with previous findings that have demonstrated RSK family members to be mediators of resistance to PI3K pathway inhibition in breast cancer, and to be capable of promoting disease progression in HNSCC specifically [35, 36].

Other studies have reported that residual mTORC1 activity following PI3K inhibition is involved with limiting its anti-tumor efficacy [49, 50]. The activation of MAPK signalling observed in our alpelisib-resistant HNSCC cell lines supports this finding, as the MAPK pathway intersects with the PI3K pathway at several downstream points that promote mTORC1 or S6 activity (Fig. 7a) [51]. As well, Chandarlapaty et al. (2011) described a direct association between inhibition of PI3K/Akt signalling and upregulation of RTKs, such as HER3 and IGF-1R [52]. The pattern of receptor upregulation/activation and intracellular signalling converging on mTORC1/S6 may be a shared feature of acquired resistance to PI3K pathway inhibition across different cancer types [12, 52]. However, the particular mechanism and mediator(s) adopted by tumor cells are likely cancer- and/or drug-specific.

Recently, PDX models have emerged as a leading preclinical platform through which to interrogate drug efficacy, interpatient response heterogeneity and, more recently, to elucidate mechanisms of drug resistance [18, 53, 54]. In our study, we confirmed in vitro findings of TYRO3 and AXL upregulation and MAPK pathway activation upon prolonged PI3Kα inhibition in a panel of 5 unique HNSCC PDX models treated for up to 100 days with alpelisib.

Based on our collective findings, pan-TAM inhibition emerges as a logical combinatorial or second-line treatment target alongside PI3Kα inhibition in HNSCC. While AXL inhibitors are already in active development owing to its identified role in drug resistance, our findings reveal its family member TYRO3 to be similarly relevant [31]. The importance of TYRO3 is particularly evident through our in vivo models, where we found TYRO3 protein expression to be significantly upregulated in alpelisib-resistant PDX tissues, whereas the changes in AXL expression were less apparent. We would therefore speculate that the use of a dual AXL/TYRO3 or pan-TAM inhibitor (e.g. LDC1267) would be more effective and durable over time [31]. To date, no-specific TYRO3 inhibitors are available. Targeting the MAPK pathway is an alternative approach, as we demonstrated with the P90RSK inhibitor BI-D1870. However, MAPK pathway inhibition has had variable efficacy to date and acquired resistance to inhibitors of the MAPK pathway has been documented, in some cases involving TAM RTKs [55, 56]. Upstream targeting of AXL and TYRO3 therefore seems to be the most logical approach. Importantly, as AXL has been identified as a drug resistance mediator to PI3K inhibition in several cancer types, pan-TAM inhibition may be a sensible approach to preventing resistance in settings even beyond HNSCC; this is a key area for future study.

## Conclusion

In summary, our findings identify TYRO3/AXL upregulation and MAPK pathway activation as a consequence of prolonged PI3Kα inhibition, both in vitro and in vivo. A therapeutic approach involving not only AXL inhibition, but pan-TAM RTK inhibition may therefore help to prevent, or at least delay, resistance to PI3K inhibitors and improve outcomes for HNSCC patients.

## Supplementary information

**Additional file 1.** Supplemental Tables & Figures. Additional Table 1. Sources and cell culture media for established HNSCC cell lines used in this study. **Additional Table 2.** Short-tandem repeat (STR) profiling results confirming matching identities of primary tumor, blood, xenograft tumors and cell lines, where available. **Additional Table 3.** Antibodies used in this study. **Additional Table 4.** Clinical features of HNSCC patients used to generate PDX models of acquired drug resistance. **Additional Fig. 1.** (A) Schematic outlining the derivation of cell line from PDX-C. (B) Phase contrast microscopy image of PDX-C cells. (C) Flow cytometry for cell surface expression of EpCAM (CD326) in PDX-C cells. Over 99% of PDX-C cells were found to be CD326-positive. **Additional Fig. 2.** Schematic outlining the development of the alpelisib-resistant HNSCC cell lines. Parental cells (Cal33 and 93-VU-147T) were treated with increasing doses of alpelisib, beginning with their IC50 values (0.5 μM for Cal33, 1.7 μM for 93-VU-147T), as previously established [20]. **Additional Fig. 3.** Relative abundance of small, medium and large sized colonies for parental and alpelisib-resistant cell lines ((A) Cal33 cells, (B) 93-VU-147T cells) when untreated, and when treated with alpelisib. Colony counts and sizes were analyzed in ImageJ version 1.52a. Briefly, RGB images of wells were converted in binary images and analyzed using the Analyze Particles feature. Colony size cutoffs were set as follows (in pixels): Small 0–100; Medium 101–500; Large > 501. **Additional Fig. 4**. (A) & (B) Immunoblot of MER-TK expression in parental and alpelisib-resistant Cal33 and 93-VU-147T cells. Short and long exposures of MER-TK blot are shown. HEK293T cells served as a positive control for MER-TK expression. **Additional Fig. 5.** Histological comparison of PDX tissues and their corresponding primary tumors (where available), stained with H&E. Scale bar represents 50 μM. **Additional Fig. 6.** Representative IHC sections showing Ki67 staining PDX tissues treated with the vehicle agent (corn oil) or alpelisib (endpoint either while still responding or treated out to the emergence of resistance). Scale bar represents 100 μM. **Additional Fig. 7.** Representative IHC sections showing AXL staining in PDX-C and PDX-E models. Quantification completed using Fiji software is shown below. ns = not significant, unpaired Student’s *t*-test. Scale bars represent 100 μM. **Additional Fig. 8**. Immunoblot of TYRO3 and AXL expression in 25 HNSCC cell lines, ordered by sensitivity to alpelisib (IC_50_ values indicated).

**Additional file 2.** Supplemental Methods & Materials.

## Data Availability

All data generated or analyzed during this study are included in this published article (and its supplementary information files).

## Abbreviations

FBS: Fetal bovine serum
FDA: Food and Drug Administration
H & E: Hematoxylin & eosin
HNSCC: Head and neck squamous cell carcinoma
IC_50_: Half-maximal inhibitory concentration values
IHC: Immunohistochemistry
MeOH: Methanol
miRNA: microRNA
MTA-1: Manual Tissue Arrayer
PDX: Patient-derived xenografts
PI3K: Phosphatidylinositol 3-kinase
RPPA: Reverse phase protein array
RTK: Receptor tyrosine kinase
SD: Standard deviation
SDS: Sodium dodecyl sulfate
Ser: Serine
siCT: Scrambled control siRNA
TAM: TYRO3, AXL, MER-TK
Thr: Threonine
Tyr: Tyrosine
