# Supplementary material for: TAM family receptors in conjunction with MAPK signalling are involved in acquired resistance to PI3Kα inhibition in head and neck squamous cell carcinoma
Source: J Exp Clin Cancer Res. 2020 Oct 15;39:217. doi: 10.1186/s13046-020-01713-9 (PMC7559997; doi:10.1186/s13046-020-01713-9)
Supplement: Supplementary file 1 — Additional file 1. Supplemental Tables & Figures. Additional Table 1. Sources and cell culture media for established HNSCC cell lines used in this study. Additional Table 2. Short-tandem repeat (STR) profiling results confirming matching identities of primary tumor, blood, xenograft tumors and cell lines, where available. Additional Table 3. Antibodies used in this study. Additional Table 4. Clinical features of HNSCC patients used to generate PDX models of acquired drug resistance. Additional Fig. 1. (A) Schematic outlining the derivation of cell line from PDX-C. (B) Phase contrast microscopy image of PDX-C cells. (C) Flow cytometry for cell surface expression of EpCAM (CD326) in PDX-C cells. Over 99% of PDX-C cells were found to be CD326-positive. Additional Fig. 2. Schematic outlining the development of the alpelisib-resistant HNSCC cell lines. Parental cells (Cal33 and 93-VU-147T) were treated with increasing doses of alpelisib, beginning with their IC50 values (0.5 μM for Cal33, 1.7 μM for 93-VU-147T), as previously established [20]. Additional Fig. 3. Relative abundance of small, medium and large sized colonies for parental and alpelisib-resistant cell lines ((A) Cal33 cells, (B) 93-VU-147T cells) when untreated, and when treated with alpelisib. Colony counts and sizes were analyzed in ImageJ version 1.52a. Briefly, RGB images of wells were converted in binary images and analyzed using the Analyze Particles feature. Colony size cutoffs were set as follows (in pixels): Small 0–100; Medium 101–500; Large > 501. Additional Fig. 4. (A) & (B) Immunoblot of MER-TK expression in parental and alpelisib-resistant Cal33 and 93-VU-147T cells. Short and long exposures of MER-TK blot are shown. HEK293T cells served as a positive control for MER-TK expression. Additional Fig. 5. Histological comparison of PDX tissues and their corresponding primary tumors (where available), stained with H&E. Scale bar represents 50 μM. Additional Fig. 6. Representative IHC sections showing [file 13046_2020_1713_MOESM1_ESM.pdf]

Additional Table 1. Sources and cell culture media for established HNSCC cell lines used in this study.

Cell Line	HPV Status	Tumour Site (if available)	Patient Information (if available)	Growth Medium	Source
**93-VU-147T**	Positive	Floor of mouth	Male, T4N2	DMEM/F12	VUMC
**Cal33**	Negative	Tongue	Male	DMEM + HI FBS + NEAA	DSMZ
**SCC15**	Negative	Tongue	Male	DMEM/F12	ATCC
**PCI6A**	Negative	—	—	DMEM/F12	University of Pittsburgh
**SCC61**	Negative	—	—	DMEM/F12	Yale
**RF37A**	Negative	—	—	DMEM/F12	University of Pittsburgh
**HSC2**	Negative	Oral cavity	Male	EMEM	
**UMSCC47**	Positive	Lateral tongue	Male, T3N1M0	DMEM/F12	University of Michigan
**UPCI:SCC154**	Positive	Oral cavity	Male, T4N2	DMEM/F12	University of Pittsburgh
**JHU029**	Negative	Oropharynx	Male, T4N0	DMEM/F12	Johns Hopkins
**RF15B**	Negative	—	—	DMEM/F12	University of Pittsburgh
**HMS001**	Positive	Oropharynx (tonsil)	Male	DMEM/F12	Harvard Medical School
**PCI6B**	Negative	Oropharynx	Male, T3N3M0	DMEM/F12	University of Pittsburgh
**D562**	Negative	Pharynx	Female	DMEM/F12	ATCC
**SCC25**	Negative	Hypopharynx	Male	DMEM/F12	ATCC
**UPCI:SCC090**	Positive	Oropharynx (tongue base)	Male, T2N0	DMEM/F12	University of Pittsburgh
**PCI13**	Negative	Oral cavity	Male, T4N1M0	DMEM/F12	University of Pittsburgh
**RF22A**	Negative	—	—	DMEM/F12	University of Pittsburgh
**JHU006**	Negative	—	—	DMEM/F12	Johns Hopkins
**Cal27**	Negative	Tongue	Male	DMEM/F12	ATCC
**SCC9**	Negative	Tongue	Male	DMEM/F12	ATCC
**JHU011**	Negative	Larynx	Male, T3N0	DMEM/F12	Johns Hopkins
**SCC4**	Negative	Tongue	Male	DMEM/F12	ATCC
**SCC2**	Positive	—	—	DMEM/F12	
**FaDu**	Negative	Hypopharynx	Male	DMEM/F12	ATCC

DMEM, Dulbecco's Modified Eagle Medium; EMEM, Eagle's Minimum Essential Medium; NEAA, no essential amino acids; VUMC, VU University Medical Center Amsterdam; DSMZ, Deutsche Sammlun von Mikroorganismen und Zellkulturen; ATCC, American Type Culture Collection

Additional Table 2. Short-tandem repeat (STR) profiling results confirming matching identities of primary tumor, blood, xenograft tumors and cell lines, where available.

Sample	Amelogenin	CSF1PO	D13S317	D16S539	D18S51	D19S433	D21S11	D2S1338	D3S1358	D5S818	D7S820	D8S1179	FGA	TH01	TPOX	vWA
**PDX-C Blood**	X,Y	10,12	11,13	11,12			28,32.2			11,11	10,10			6,9.3	8,11	16,16
**PDX-C Primary**	X,Y	10,12	11,13	11,12			28,32.2			11,11	10,10			6,9.3	8,11	16,16
**PDX-C C4**	X,Y	10,12	11,13	11,12	11,13	14,14	28,28	17,18	15,15	11,11	10,10	13,13	25,25	6,9.3	8,11	16,17
**PDX-C C5**	X,Y	10,12	11,13	11,12			28,32.2			11,11	10,10			6,9.3	8,11	16,17
**PDX-C B2**	X,Y	10,12	11,13	11,12			28,28			11,11	10,10			6,9.3	8,11	16,17
**PDX-C B3**	X,Y	10,12	11,13	11,12	11,13	14,14	28,28	17,18	15,15	11,11	10,10	13,13	25,25	6,9.3	8,11	16,17
**PDX-C B4**	X,Y	10,12	11,13	11,12	11,13	14,14	28,32.2	17,18	15,15	11,11	10,10	13,13	25,25	6,9.3	8,11	16,17
**PDX-C Cell Line**	X,Y	12,12	11,13	11,12	11,13	14,14	28,32.2	17,18	15,15	11,11	10,10	13,13	25,25	6,9.3	8,11	16,17
**PDX-E Primary**	X,X	12,12	12,12	9,9			30,30,2			12,13	11,11			7,9.3	8,11	17,17
**PDX-E C3**	X,X	12,12	12,12	9,9	10,18	12,16	30,30,2	19,19	17,17	12,13	11,11	13,13	22,23	7,9.3	11,11	17,17
**PDX-E C4**	X,X	12,12	12,12	9,9			30,30,2			12,13	11,11			7,9.3	11,11	17,17
**PDX-E B2**	X,X	12,12	12,12	9,9	10,18	12,16	30,30,2	19,19	17,17	12,13	11,11	13,13	22,23	7,9.3	8,11	17,17
**PDX-E B3**	X,X	12,12	12,12	9,9			30,30,2			12,13	11,11			7,9.3	8,11	17,17
**PDX-E B4**	X,X	12,12	12,12	9,9	10,18	12,16	30,30,2	19,19	17,17	12,13	11,11	13,13	22,23	7,9.3	8,11	17,17

Additional Table 3. Antibodies used in this study.

Antibody	Company	Catalogue Number	Dilution
**pAkt (T308)**	CST	4056	1:1000
**pAkt (S473)**	CST	9271	1:1000
**Akt (pan)**	CST	4685	1:1000
**pERK1/2 (T202/Y204)**	CST	4370	1:1000
**ERK1/2**	CST	4696	1:1000
**pP90RSK (S380)**	CST	11989	1:1000
**P90RSK (pan)**	CST	9355	1:1000
**PARP**	BD Pharmingen	556494	2ug/mL
**Cleaved PARP**	CST	5625	1:1000
**TYRO3**	CST	5585	1:1000
**AXL**	CST	8661	1:1000
**MER-TK**	abcam	ab52968	1/2000
**GAB2**	CST	3239	1:1000
**pMEK1 (S298)**	CST	9128	1:1000
**MEK1**	CST	2352	1:1000
**pS6 (S235/6)**	CST	4858	1:1000*
**S6**	CST	2217	1:1000*
**EGFR**	CST	4267	1:2000
**MER-TK^{+}**	abcam	ab52968	1/500
**TYRO3^{+}**	BETHYL Laboratories Inc.	IHC-00410	1:100-1:500
**α-tubulin**	CST	2125	1:1000

CST, Cell Signaling Technology
* primary antibody incubation: 1hr at room temperature
$^{+}$ for IHC

Additional Table 4. Clinical features of HNSCC patients used to generate PDX models of acquired drug resistance.

PDX ID	Gender	Age	TNM Stage T	TNM Stage N	TNM Stage M	Disease Site	Subsite	HPV Status	Smoking History	Alcohol Consumption	Recurrent
PDX-A	M	72	T2	N2b	M0	Lip & Oral Cavity	Tongue	nt	Ex-smoker	Non-drinker	No
PDX-B	M	60	T3	N2c	M0	Oropharynx	Base of Tongue	+	Non-smoker	Non-drinker	Yes
PDX-C	M	44	T2	N1	M0	Lip & Oral Cavity	Tongue	-	Non-smoker	Non-drinker	No
PDX-D	F	87	T2	N2b	M0	Lip & Oral Cavity	Tongue	nt	Non-smoker	Non-drinker	No
PDX-E	M	63	T4	N2b	M0	Hypopharynx	Piriform Sinus	-	NA	NA	NA

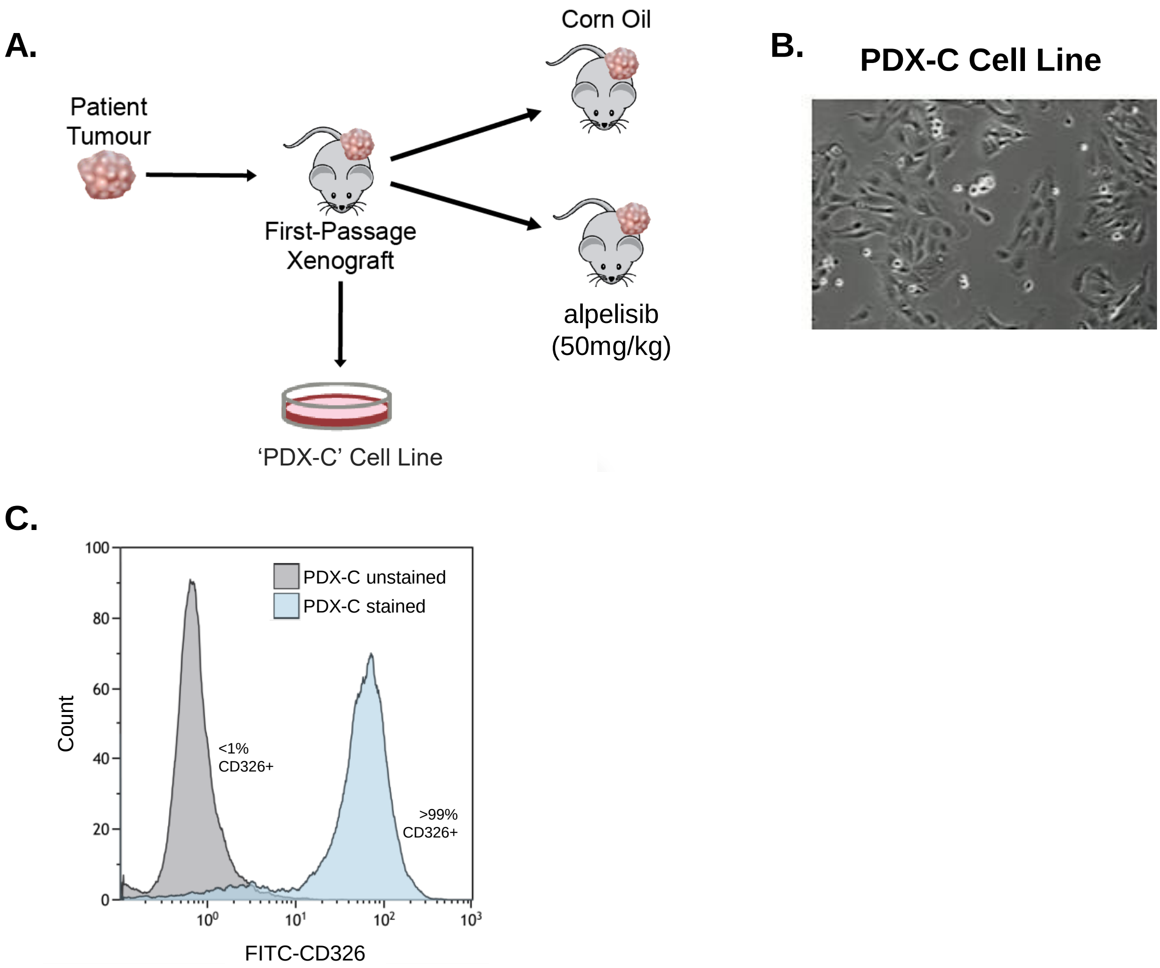

Additional Fig. 1. (A) Schematic outlining the derivation of cell line from PDX-C. **(B)** Phase contrast microscopy image of PDX-C cells. **(C)** Flow cytometry for cell surface expression of EpCAM (CD326) in PDX-C cells. Over 99% of PDX-C cells were found to be CD326-positive.

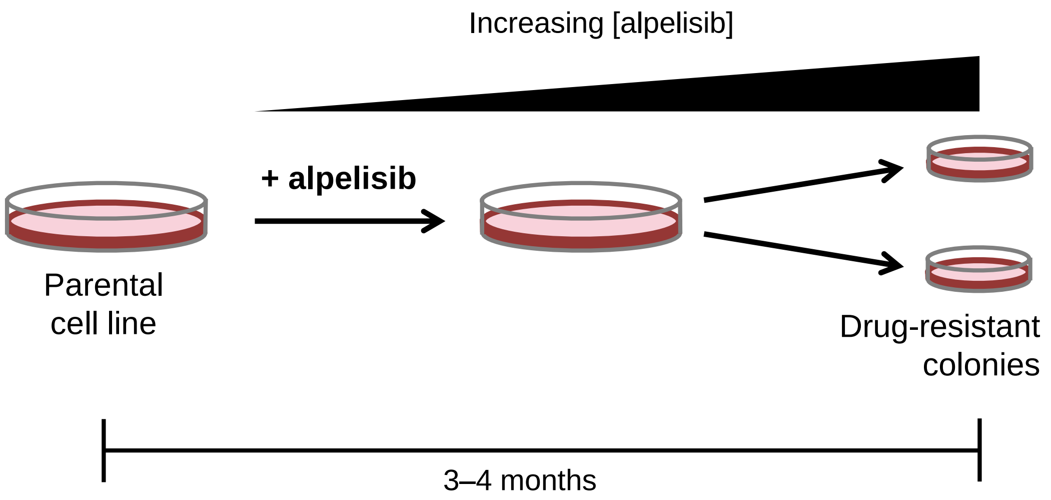

Additional Fig. 2. Schematic outlining the development of the alpelisib-resistant HNSCC cell lines. Parental cells (Cal33 and 93VU-147T) were treated with increasing doses of alpelisib, beginning with their IC_{50} values (0.5μM for Cal33, 1.7μM for 93VU-147T), as previously established (20).

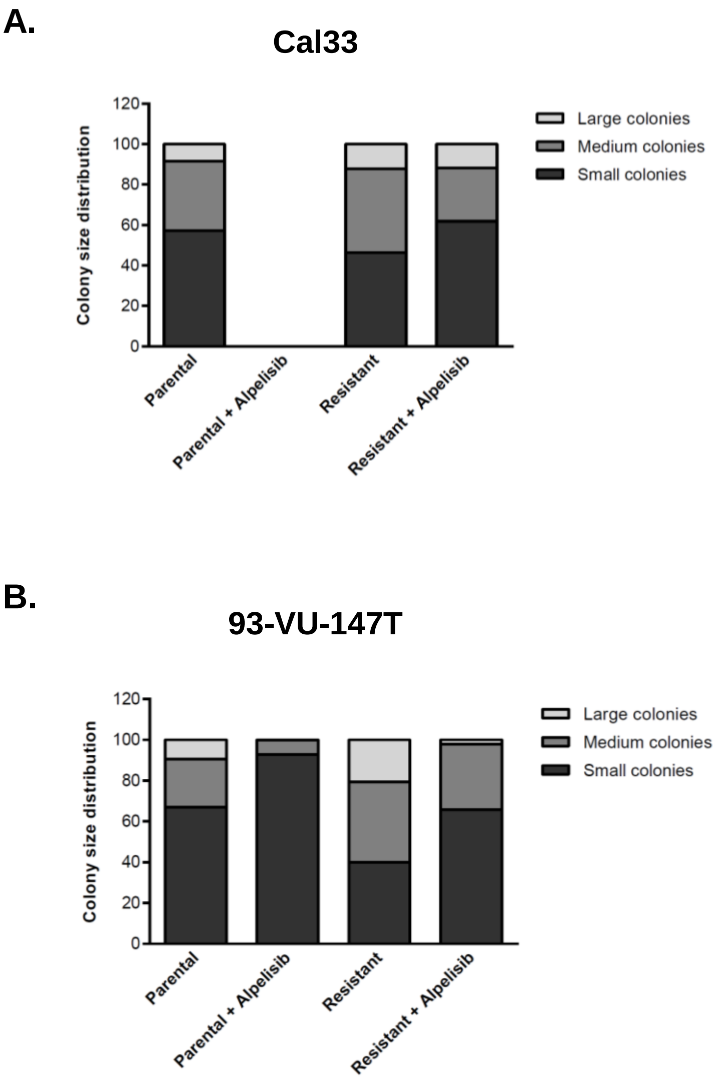

Additional Fig. 3. Relative abundance of small, medium and large sized colonies for parental and alpelisib-resistant cell lines (**(A)** Cal33 cells, **(B)** 93-VU-147T cells) when untreated, and when treated with alpelisib. Colony counts and sizes were analyzed in ImageJ version 1.52a. Briefly, RGB images of wells were converted in binary images and analyzed using the Analyze Particles feature. Colony size cutoffs were set as follows (in pixels): Small 0-100; Medium 101-500; Large $\geq$ 501.

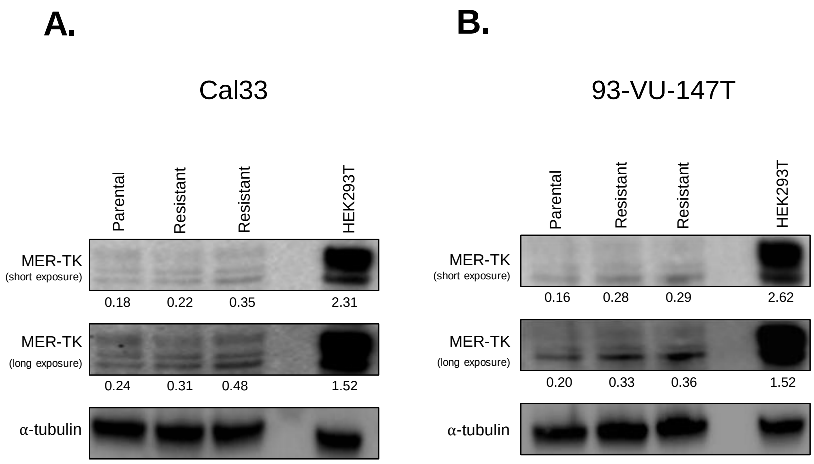

Additional Fig. 4. (A) & (B) Immunoblot of MER-TK expression in parental and alpelisib-resistant Cal33 and 93-VU-147T cells. Short and long exposures of MER-TK blot are shown. HEK293T cells served as a positive control for MER-TK expression.

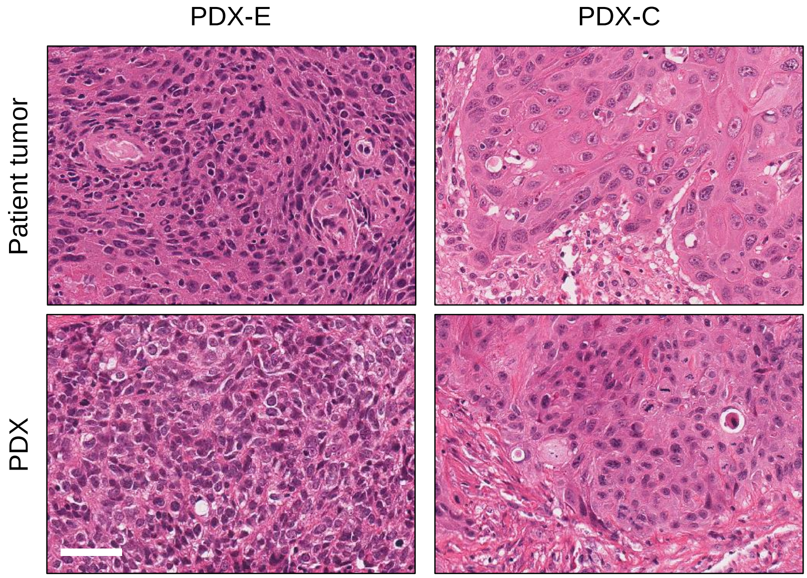

Additional Fig. 5. Histological comparison of PDX tissues and their corresponding primary tumors (where available), stained with H&E. Scale bar represents 50μM.

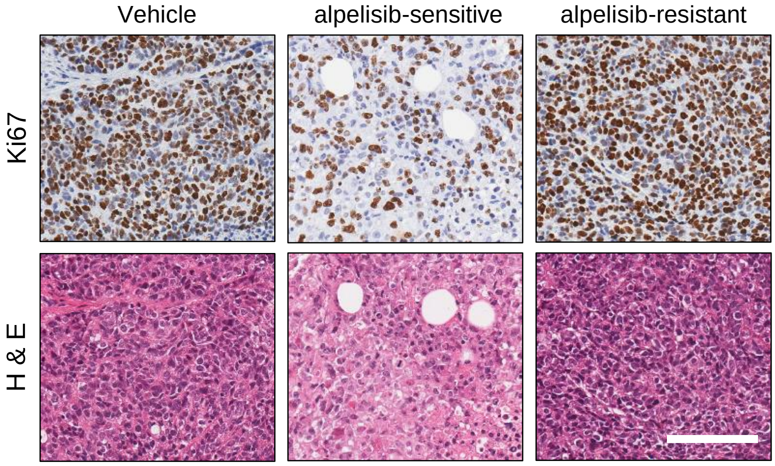

Additional Fig. 6. Representative IHC sections showing Ki67 staining PDX tissues treated with the vehicle agent (corn oil) or alpelisib (endpoint either while still responding or treated out to the emergence of resistance). Scale bar represents 100μM.

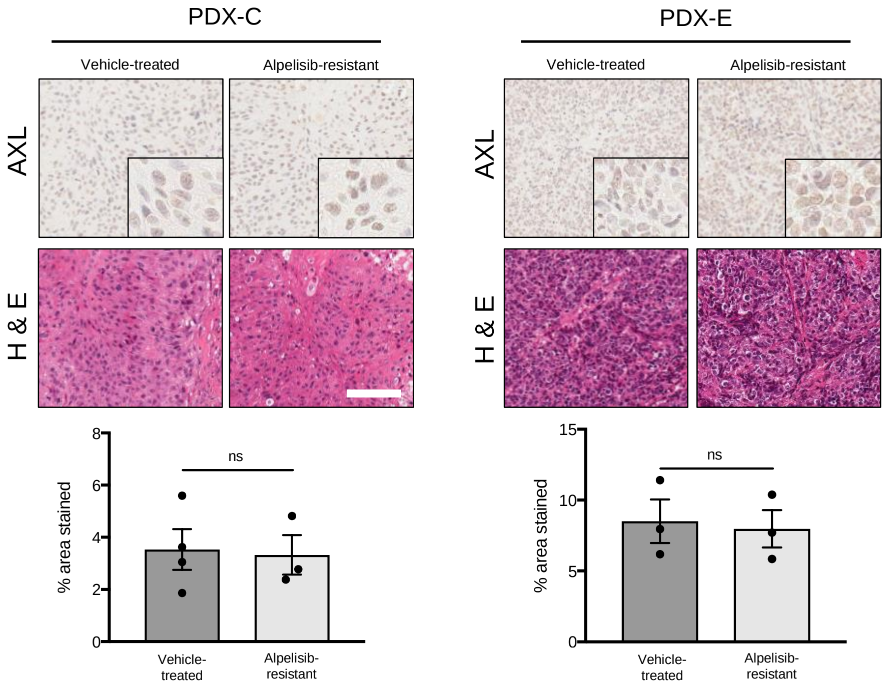

Additional Fig. 7. Representative IHC sections showing AXL staining in PDX-C and PDX-E models. Quantification completed using Fiji software is shown below. ns = not significant, unpaired Student's *t*-test. Scale bars represent 100μM.

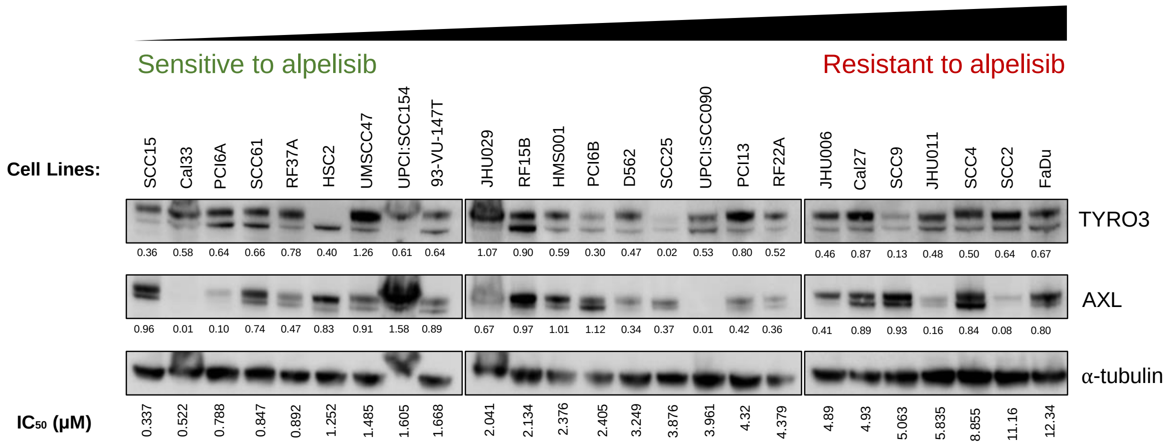

Additional Fig. 8. Immunoblot of TYRO3 and AXL expression in 25 HNSCC cell lines, ordered by sensitivity to alpelisib (IC_{50} values indicated).

Additional Fig. 8
KM Ruicci *et al.*, 2020
