# Supplementary material for: TAM family receptors in conjunction with MAPK signalling are involved in acquired resistance to PI3Kα inhibition in head and neck squamous cell carcinoma
Source: J Exp Clin Cancer Res. 2020 Oct 15;39:217. doi: 10.1186/s13046-020-01713-9 (PMC7559997; doi:10.1186/s13046-020-01713-9)
Supplement: Supplementary file 2 — Additional file 2. Supplemental Methods & Materials. [file 13046_2020_1713_MOESM2_ESM.docx]

**Supplemental Methods & Materials**

*Quantitative Real-Time PCR (qRT-PCR)*

qRT-PCR analysis was completed as described previously^17^. Primers (5’ to 3’): AXL (F -AGGGCCGGGGACAGC, R -AGCCTGCGTGCCCCT), TYRO3 (F -CCGCCGCAGGTCTGAAG, R -ACCCACTGGATGTCAGGCTC), β-actin (F - AGAGCTACGAGCTGCCTGAC, R - AGCACTGTGTTGGCGTACAG).

*Establishment of patient derived xenografts*

Fresh surgical HNSCC specimens were received from consenting patients with primarily diagnosed or recurrent HNSCC who underwent surgery at the London Health Sciences Centre or Princess Margaret Cancer Centre between 2009 and 2014 under a University Health Network Research Ethics Board approved protocol (REB #12-5639). Specimens were received within 0.5−24hrs of surgery and kept at 4°C in PBS until engraftment no later than 24hrs post-resection. Tumors were divided into ~1mm^3^ pieces and implanted subcutaneously into the flank region of NOD/SCID/IL2Rγ^-/-^ (NSG) male mice. Once tumors reached 1−1.5cm in size, mice were sacrificed and tumors were dissected from the flank, dissociated in culture medium containing collagenase/hyaluronidase and DNASE 1 and passaged subcutaneously into 10 mice per tumor model (minimum 100,000 cells/mouse) in 1:1 matrigel/PBS. Once tumors were palpable, measurements with calipers began. Tumors were classified as HPV-positive using immunohistochemistry (IHC) for p16.

Once tumor volumes reached 80−120mm^3^ mice were randomized to either daily (5x/week) BYL719 (Novartis; 50mg/kg) by oral gavage or a vehicle control (corn oil). Mice were maintained until tumors reached a maximum size of 1.5 cm in diameter or an alternative humane endpoint was reached as stated in the animal protocol. Animals were observed daily for their overall health. Mice were evaluated for tumor size and body weight every 2−4 days. Individual tumor volumes were calculated using the formula: [length x (width)^2^] x 0.52. Where possible, STR profiling was used to confirm matching identifies of primary tumors, xenograft tumors, patient blood and PDX-derived cell lines where available (**Supp. Table 2**).
